# Human TorsinA can function in the yeast cytosol as a molecular chaperone

**DOI:** 10.1042/BCJ20170395

**Published:** 2017-10-05

**Authors:** Ilectra Adam, Lyne Jossé, Mick F. Tuite

**Affiliations:** Kent Fungal Group, School of Biosciences, University of Kent, Canterbury, Kent CT2 7NJ, U.K.

**Keywords:** Hsp104, molecular chaperone, prion, *Saccharomyces cerevisiae*, TorsinA

## Abstract

TorsinA (TorA) is an AAA+ (ATPases associated with diverse cellular activities) ATPase linked to dystonia type 1 (DYT1), a neurological disorder that leads to uncontrollable muscular movements. Although DYT1 is linked to a 3 bp deletion in the C-terminus of TorA, the biological function of TorA remains to be established. Here, we use the yeast *Saccharomyces cerevisiae* as a tractable *in vivo* model to explore TorA function. We demonstrate that TorA can protect yeast cells against different forms of environmental stress and show that in the absence of the molecular disaggregase Hsp104, TorA can refold heat-denatured luciferase *in vivo* in an ATP-dependent manner. However, this activity requires TorA to be translocated to the cytoplasm from the endoplasmic reticulum in order to access and process cytoplasmic protein aggregates. Furthermore, mutational or chemical inactivation of the ATPase activity of TorA blocks this activity. We also find that TorA can inhibit the propagation of certain conformational variants of [*PSI*^+^], the aggregated prion form of the endogenous Sup35 protein. Finally, we show that while cellular localisation remains unchanged in the dystonia-linked TorA mutant ΔE302-303, the ability of this mutant form of TorA to protect against cellular stress and to facilitate protein refolding is impaired, consistent with it being a loss-of-function mutation.

## Introduction

TorsinA (TorA) was originally identified as a protein connected with human dystonia type 1 (DYT1), a congenital movement disorder [1,2], and thus, its biological function has since attracted much interest. DYT1 is associated with a three-base pair (ΔGAG) deletion that removes one of a pair of glutamic acid residues (Glu-302/303) from near the C-terminus of TorA [3–5]. However, the apparent association of TorA with several different cellular compartments and cofactors and its implication in multiple cellular roles [6] continues to confuse researchers as efforts are made to understand how it contributes to this neurological disorder.

TorA is targeted to the lumen of endoplasmic reticulum (ER) by a 20 amino acid, N-terminal signal sequence [7–9], where it is glycosylated. In the ER, TorA is anchored to the ER membrane via a short hydrophobic domain that is exposed by cleavage of the signal sequence [3,10,11]. Two cysteine residues in this N-terminal hydrophobic domain act as a proteolytic cleavage site, and ER stress triggers this cleavage leading to the mobilisation of TorA from the ER membrane [12]. Some of the functions of TorA, however, have been linked to membrane attachment and assembly due to its interaction with the nuclear envelope protein LAP1 (lamina-associated polypeptide) and the ER membrane-associated LULL1 (luminal domain-like LAP1), two cofactors that also regulate TorA ATPase activity [4,13]. TorA is also associated with anterograde cytoplasmic transport via binding with the cytoplasmic kinesin light complex (KLC1) [14], while a recent study in *Drosophila* has implicated TorA in regulating cellular lipid metabolism [15]. All these studies suggest that the biological role(s) of TorA are defined to some extent by its relevant subcellular location.

TorA is a member of the superfamily of AAA+ (ATPases associated with diverse cellular activities) ATPases that are associated with diverse cellular activities [10,16], although TorA is the only member that is ER-associated. Several studies have suggested a role for TorA in protecting eukaryotic cells against protein misfolding, aggregation and proteotoxicity [17–21]. How this protective role is achieved remains to be established, but possibly relates to a chaperone-like activity. This possibility emerged from the realisation that TorA has a significant level of amino acid sequence identity with ClpB [3,16], a member of the AAA+ superfamily that acts as a molecular chaperone in bacteria. Subsequently, studies have shown that TorA can reactivate heat-denatured luciferase *in vitro* [22], but as yet there has been no report of such protein disaggregase activity *in vivo*. Elevated levels of TorA can certainly protect cells against various forms of stress, such as heat shock and oxidative stress, consistent with a chaperone-like function *in vivo* [19–21]. That TorA co-localises with the Hsp70/40 chaperones in Lewy bodies to suppress α-synuclein aggregation suggests a putative interaction between TorA and the endogenous chaperone network to target misfolded proteins [17].

To explore the function and mechanism of action of TorA, we have used the yeast *Saccharomyces cerevisiae* as an *in vivo* test bed. *S. cerevisiae* is an attractive unicellular model in which to explore the fundamental aspects of cellular mechanisms and has been, for example, successfully employed in the study of certain proteopathies [23–25]. A previous study by Valastyan and Linquist [26] found that directing TorA to the ER of yeast was not detrimental to growth, but they were unable to demonstrate an impact on either protein folding or secretion albeit with a limited set of test substrates. A subsequent study demonstrated that TorA can be directed to the yeast ER using the native human TorA signal sequence, and using this system, the authors were able to demonstrate the essential role played by resident ER chaperones, specifically BiP, in TorA biogenesis [27].

*S. cerevisiae* does not encode an orthologue of TorA, but it does have many AAA+ ATPases including Hsp104, a mainly cytoplasmic protein that has been phylogenetically linked to the ClpB/Hsp100 family of molecular chaperones. Hsp104 has no close mammalian orthologue, but like other members of the AAA+ ATPase family it assembles into a homo-hexameric ring-like structure [28,29]. The major cellular role of Hsp104 is a protective one, ensuring the recovery from heat shock during which the protein's disaggregase activity facilitates the reactivation of misfolded proteins. This function is coupled with ATP hydrolysis [30–34], itself driven by various regulatory co-chaperones. While TorA contains one nucleotide domain [NBD (nucleotide-binding domain)] with motifs that are important for ATP binding (Walker A) and hydrolysis (Walker B) [35,36], Hsp104 has two such domains, NBD1 and NBD2. A series of point mutations have been described in the NBDs that impair nucleotide-binding activity of both Hsp104 [37,38] and TorA [39,40]. The ATPase activity of Hsp104 can also be inhibited *in vivo* by growing cells in millimolar concentrations of the protein-denaturing agent guanidine hydrochloride (GdnHCl) [41–43], but any effect of GdnHCl on TorA has not been reported.

While TorA and Hsp104 differ in their main pattern of cellular localisation and the number of NBDs, they clearly share many structural and functional properties. For example, they both protect cells against different forms of stress — albeit in different contexts — so determining whether they can functionally substitute for each other *in vivo* would shed new light on the cellular role of TorA.

## Materials and methods

### Plasmid construction

Plasmid pUKC2752 was generated by cloning TorA cDNA as a *Bam*HI–*Sal*I fragment into the *URA3-*based expression plasmid pBEVY-U, which has the constitutive *ADH1* promoter [44]. Single residue TorA mutants were constructed with the QuikChange Lightning Site-Directed Mutagenesis Kit (Agilent Technologies) using pUKC2752 as the template. Plasmid pUKC2753 was created using pUKC2752 as a template to amplify ΔN-TorA (TorsinA lacking the N-terminal signal sequence) as a *Bam*HI–*Sal*I fragment and to ligate into pBEVY-U. Versions of TorA and ΔN-TorA that were N-terminally tagged with GFP were created using Gateway Cloning Technology (Invitrogen) with the BP and LR recombination reactions being carried out essentially according to the manufacturer's instructions. The resulting plasmids were designated pUKC2774 (TorA-GFP) and pUKC2776 (ΔN-TorA-GFP). The oligonucleotide primers used for mutagenesis are listed in Supplementary Table S1.

A plasmid based on pBEVY-U and expressing the wild-type *S. cerevisiae HSP104* gene (designated pUKC2751) was previously made in this laboratory. The plasmid pJK59 (also called pPS1622) encodes Sec63-GFP, a fusion of the S65T/V163A double mutant of GFP to the C-terminus of Sec63, under the SEC63 promoter, and was a gift from Pamela Silver (AddGene plasmid # 8854).

### Yeast strains

The *S. cerevisiae* strains BY4741 (*MATa his3Δ1 leu2Δ0 met15Δ0 ura3Δ0*), YJW532 Δhsp104 (*MATa ade1-14 his3-11,15 leu2-3,112 ura3-1 trp1-1 can1-100, hsp104::HIS3*, pRS316HSP104) and 74D-694 (*MATα ade1-14 trp1-289 his3Δ-200 ura3-52 leu2-3,112*) were used in the present study. BY4741 was used for the luciferase and stress assays (see below) as well as the TorA localisation studies, while [*psi*^−^] and [*PSI*^+^] versions of 74D-694 strains and the YJW532 strain were used for the prion assays.

### Galactose-induced gene expression

Typically, yeast cells transformed with *URA3*-based plasmids pUKC2762 or the backbone plasmid pYES2 were grown overnight at 30°C in a yeast nitrogen base (YNB)-based pre-induction synthetic complete medium (0.67% YNB, supplemented with amino acid plus base mix without uracil, Formedium) with 2% raffinose. Cells were then harvested and transferred to induction medium (the same synthetic complete medium lacking uracil but with 2% galactose rather than raffinose) and incubated for a further 16–18 h before being collected for protein extraction.

### Western blot analysis

Cell-free extracts were prepared from logarithmic phase cultures as described by von der Haar [45] and were analysed by SDS–PAGE using either NuPAGE 10% Bis–Tris gels or 4–20% Tris–Glycine gradient gels (Invitrogen). Proteins were then transferred onto the PVDF membrane (Roche) and probed with either an anti-TorA polyclonal antibody (gift from Dr Lisa Swanton, University of Manchester), or an anti-Hsp104 polyclonal antibody (from our own laboratory), or an anti-Sup35 polyclonal antibody (MT50 from our laboratory) or an anti-phosphoglycerate kinase (PGK) polyclonal antibody (MT102 from our laboratory). Anti-rabbit HPR-conjugated antibodies (Sigma) were used as a secondary antibody in the ECL analysis. EndoH (New England Biolabs) digests were performed according to the manufacturer's instructions prior to electrophoresis.

### *In vivo* luciferase refolding assay

The *in vivo* luciferase refolding assay [46] was adapted from Zenthon et al. [47] and used the pGPDLuxAB(*HIS3*) plasmid which encodes a heat-sensitive Vibrio harveyi luciferase (gift from Susan Lindquist, Whitehead Institute for Biomedical Research, MIT). A single fresh colony of the relevant strain transformed with pGPDLuxAB(*HIS3*) was grown overnight in 5 ml of selective medium (0.67% YNB and 2% glucose supplemented with amino acid mixture without histidine, Formedium). Cells were then diluted to *A*_600_ = 0.2 and grown on at 30°C to an *A*_600_ = 0.4. At this point, the endogenous luciferase activity was measured using a BMG Labtech FLUOstar OPTIMA plate reader using 300 µl of cell suspension per reading. Decanal (5 µl, Sigma–Aldrich) was added to each cell sample giving a final concentration of ∼90 mM. Luminescence was immediately read to give the 100% luciferase activity value. The rest of the culture was transferred to 46°C for 12 min, then cycloheximide was added to a final concentration of 10 µg/ml and the cultures were incubated at 46°C for a further 12 min at which point luciferase activity in 300 µl of cells was measured as before. The cells were then returned to 30°C to recover, and luciferase activity was measured in 300 µl samples every 30 min for periods up to 4 h. The percentage of refolded luciferase was calculated based on the 100% luciferase activity value. Three independent samples were taken for each time point for each strain/condition and the averages were calculated.

### Cell stress assays

Cell stress assays were performed starting with cells in logarithmic phase (absorbance *A*_600_ ∼0.2) and growth was quantified by measuring the resulting absorbance at *A*_600_ for 18–22 h using a BMG Labtech FLUOstar OPTIMA plate reader. For heat shock, cells were first pre-incubated at 37°C for 1 h followed by 2 h heat shock at 47°C. In some experiments, 3 mM GdnHCl was added prior to the shift to 47°C. For ethanol stress, a final concentration of 15% (v/v) ethanol was added to the cultures and growth was monitored for 22 h.

### Cell imaging

Plasmids expressing green fluorescent protein (GFP) fusions with Sec63, Hsp104 and TorA and their respective mutants were individually transformed into both the BY4741 strain and a Δ*hsp104* derivative of this strain. Protein localisation assays were performed in logarithmic phase cells (*A*_600_ ∼0.4–0.6) and visualised using the GFP excitation filter on an Olympus 1 × 81 fluorescent microscope with a Hamamatsu Photonics Orca AG cooled CCD camera. The resulting images were then processed using the CellR software (Olympus).

To study the impact of tunicamycin on localisation of GFP-tagged TorA in yeast, early logarithmic phase cells (*A*_600_ ∼0.4–0.6) were first exposed to tunicamycin (10 µg/ml final concentration; Sigma, dissolved in DMSO) for 2 h at 30°C with shaking at 200 rpm. The cells were then visualised using a fluorescent microscope as described above. Tunicamycin treatment was also studied in pre-heat-shocked cells at 37°C for 1 h prior to the addition of tunicamycin.

### Plasmid shuffling and phenotyping analysis

Plasmid pAG425GPD-ccdB-TorA-EGFP-*LEU2* expressing a TorA-GFP fusion (generated by Susan Lindquist's Laboratory, obtained from Addgene #14202) was introduced into a derivative YJW532 strain that carries an *hsp104::kanMX* disruption and a single-copy *URA3*-based plasmid pRS316 [48] expressing the *HSP104* gene and designated pRS318*-URA3-HSP104*. This strain, a gift from Lev Osherovich and Jonathan Weissman (UCSF), and both plasmids were selected for on -leu- and -ura YNB-based synthetic medium as appropriate. Ura^−^ cells lacking the pRS316*-URA3-HSP104* plasmid were selected for by growing the double transformants overnight at 30°C in synthetic -leu/-ura medium followed by plating on YEPD supplemented with 5-fluoroorotic acid (5-FOA; 1 mg/ml). The resulting Leu^+^ Ura^−^ cells had lost the pRS316-*URA3*-*HSP104* plasmid. Further selection on -leu medium was used to identify strains that retained the pAG425GPD-ccdB-EGFP-*LEU2* plasmid. The desired Leu^+^ Ura^−^ colonies were streaked on 1/4 YEPD with and without 3 mM GnHCl and incubated for 2 days. The [*PSI*^+^]-associated phenotype was scored based on suppression or not of the *ade1-14* mutation; [*PSI*^+^] cells give rise to white colonies while [*psi*^−^] cells give rise to red Ade^−^ colonies.

### Statistical analysis

Two-way ANOVA was used for statistical analysis, using Minitab software, with the threshold for statistical significance difference taken at a 95% confidence interval. Tukey's HSD *post hoc* test was used to determine significant differences.

## Results

### The native signal sequence of TorA targets the protein to the yeast ER

**Figure 1.**
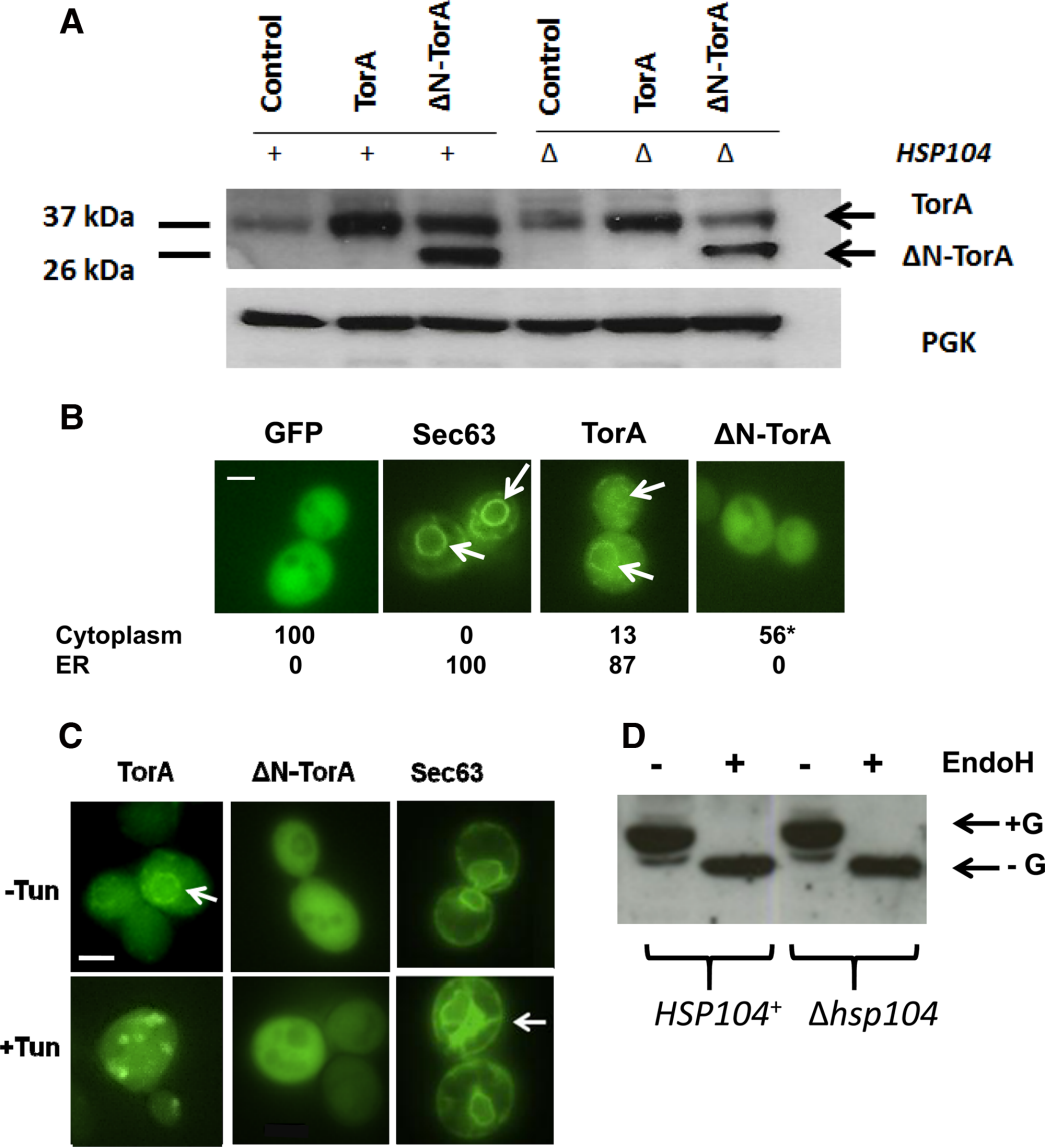
Localisation and post-translational modification of human TorA expressed in *S. cerevisiae*. (**A**) Western blot analysis using an anti-TorA antibody of total protein extracts from cells expressing either full-length TorA (TorA) or a version lacking the N-terminal signal sequence (ΔN-TorA). The control shown is cells carrying the plasmid pBEVY-U (control). Synthesis in the BY4741 parent strain (*HSP104*^+^) or BY4741 carrying a deletion of the *HSP104* gene (Δ*hsp104*) are shown. PGK was used as the protein loading control. (**B**) Localisation of TorA-GFP and ΔN-TorA-GFP fusion proteins in yeast compared with a Sec63-GFP fusion protein. The white arrows indicate localisation in the ER, and the figures are the percentage of cells (*n* = 200) showing localisation to either the ER or the cytoplasm. The magnification used was ×60. *NB*: 44% of the ΔN-TorA-GFP-expressing cells showed predominantly cell membrane-associated foci (not shown). (**C**) Localisation of TorA-GFP and ΔN-TorA-GFP in cells grown in the presence (+Tun) or absence (−Tun) of tunicamycin (10 μg/ml) at 30°C. The magnification used was ×60 and the size bar is 2 μm. (**D**) Western blot analysis of TorA in protein extracts prepared from a wild type (*HSP104*^+^) and Δ*hsp104* strains expressing TorA. Cells were either treated with EndoH (+EndoH) or not (−EndoH). The glycosylated form (+G) and non-glycosylated form (−G) of TorA are indicated.

To study the cellular function of TorA, we first needed to be able to direct it to different subcellular compartments in yeast. Two plasmids were constructed, pUKC2752 constitutively expressing the full-length wild-type TorA with its 20 residue N-terminal signal sequence for ER localisation, and a second, pUKC2753 constitutively expressing a truncated form of TorA lacking the signal sequence (designated ΔN-TorA). Both forms were expressed as confirmed by western blotting with the reduced molecular mass of ΔN-TorA consistent with a non-ER-associated and hence non-glycosylated form of TorA (Figure 1A). An endogenous protein of the same approximate molecular mass as TorA was also observed, but its identity remains to be established.

To confirm their respective localisation, GFP was fused to the C-terminus of TorA and ΔN-TorA proteins to generate plasmids pUKC2774 and pUKC2776, and the localisation of the fusion proteins was assessed by fluorescence microscopy and comparing to Sec63, an ER membrane-associated protein. The expressed TorA-GFP showed a localisation pattern in 87% of the 200 cells examined that was consistent with its localisation to the ER, i.e. it mirrored the observed Sec63-GFP localisation pattern (Figure 1B). The remaining 17% of cells showed an apparent cytoplasmic localisation for TorA-GFP. There was no evidence of nuclear localisation. As expected, no ΔN-TorA-GFP was detected in the ER, but predominantly in the cytoplasm (56% of the 200 cells examined) and the remainder as foci associated with the cell membrane.

If TorA was localised to the ER as the GFP studies suggest, we would expect the protein to be glycosylated as the protein has two Asn-linked glycosylation sites at residues 143 and 158. To confirm that the expressed TorA was N-linked glycosylated, the TorA and the ΔN-TorA-expressing cells were grown in the presence of tunicamycin, an inhibitor of N-linked glycosylation in yeast [49]. In the tunicamycin-treated cells, TorA-GFP was now evident as cytoplasmic inclusions (Figure 1C). This behaviour could be explained either as a result of misfolded non-glycosylated TorA being returned to the cytoplasm by the ERAD pathway or by failure of TorA to translocate into the ER as a consequence of induction of the unfolded protein response [50]. Tunicamycin had no impact on the localisation or cytoplasmic form of ΔN-TorA consistent with it not entering the ER, while Sec63 remained ER membrane-associated. The nature of the TorA glycoforms was confirmed using digestion with EndoH, an enzyme that cleaves asparagine-linked mannose-rich oligosaccharides from glycoproteins [51], and this resulted in a reduced molecular mass for TorA consistent with full de-glycosylation (Figure 1D).

Although Valastyan and Lindquist [26] reported that TorA could only be directed to the yeast ER using a yeast (Kex2) ER localisation sequence, our data are consistent with those of Zacchi et al. [27], namely that human TorA can localise to the yeast ER via its native signal sequence. Our data also show that in the absence of this signal sequence, TorA is unable to enter the ER and locates primarily to the cytoplasm.

### TorA protects yeast cells against environmental stress in an ATPase-dependent manner

The cytoplasmically located AAA+ ATPase, Hsp104, that shows sequence homology to TorA has a key role in tolerance to various forms of environmental stress in yeast [31]. To explore whether TorA was able to functionally replace Hsp104 to protect cells against such environmental stress, we examined the response of the strain BY4741 carrying a *Δhsp104* deletion to heat stress. A previous study reported that expression of TorA in wild-type (*HSP104*^+^) yeast had no effect on how the cells responded to a mild heat stress (39°C) [27]. Consequently, we conducted experiments in absence of Hsp104 that is essential for the acquisition of thermotolerance in yeast [30] and at higher temperatures. Expressing TorA or ΔN-TorA in the *Δhsp104* mutant did not negatively effect on levels of expression of the mature TorA protein, although the levels of ΔN-TorA were slightly reduced in the *Δhsp104* mutant (Figure 1A).

**Figure 2.**
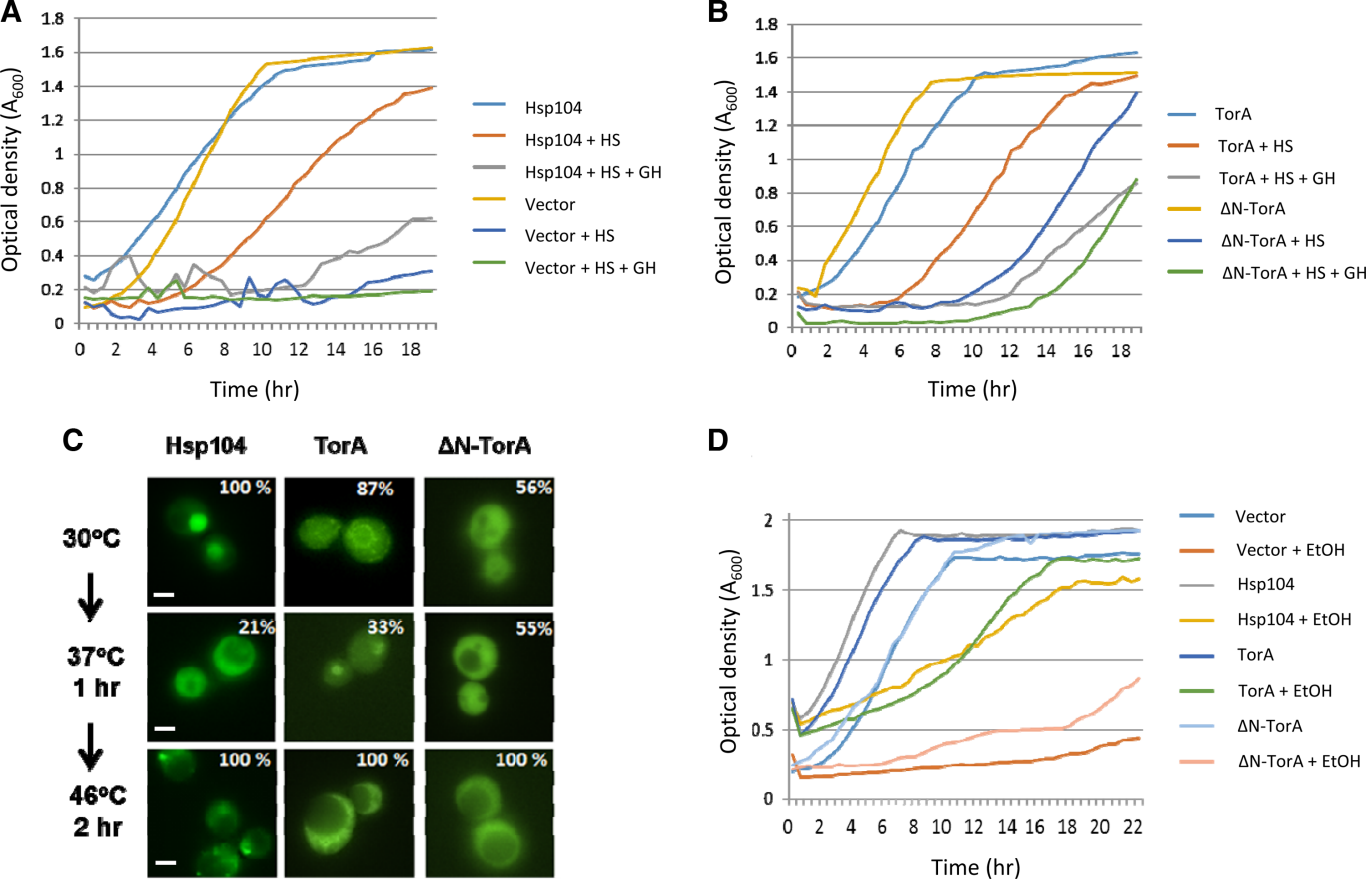
TorA can functionally replace Hsp104 in providing protection against thermal and chemical (ethanol) stress. (**A**) Thermal stress assay. Shown are growth profiles of a Δ*hsp104* strain continuously measuring culture density at 30°C over a period of 18 h. Cells either contained the plasmid pBEVY-U (Vector) or plasmid pUKC2751 constitutively expressing the *HSP104* gene under the control of the *ADH1* promoter (Hsp104). HS (heat shock), growth after exposure to 37°C for 1 h followed by 2 h at 47°C. HS + GH, as for HS except guanidine hydrochloride (GH) was added to 3 mM before the shift to 47°C. (**B**) As for (**A**) except the Δ*hsp104* strain was transformed with a plasmid expressing either TorA or ΔN-TorA as indicated. (**C**) The effect of heat stress on the localisation of Hsp104-GFP, TorA-GFP and ΔN-TorA-GFP fusion proteins in yeast compared with a Sec63-GFP fusion protein. The figures are the percentage of cells (*n* = 200) with the localisation pattern shown. The magnification used was ×60 and the size bar is 2 μm. (**D**) Growth of the Δ*hsp104* strain in the presence or absence of 15% (v/v) ethanol. The strain was either transformed with pBEVY-U (vector) or plasmids constitutively expressing Hsp104, TorA or ΔN-TorA. Growth was monitored as in (**A**) and (**B**).

TorA, ΔN-TorA or wild-type Hsp104 was expressed in the *Δhsp104* strain and compared with the vector alone as the control, under the following assay conditions. Cells were pre-incubated at 37°C for 1 h and then subjected to a 2 h heat shock at 47°C. After returning the heat-shocked cells to 30°C, absorbance was monitored at *A*_600_ continuously for a further 18 h. The *Δhsp104* cells showed no growth even after 18 h, whereas the same strain expressing wild-type Hsp104 recovered after a lag phase of ∼6 h albeit with a slightly reduced doubling time (Figure 2A). Expression of TorA in the same *Δhsp104* mutant restored growth post heat shock to a similar extent to the Hsp104 control with a 6 h lag phase and a slightly reduced doubling time (Figure 2B). For the ΔN-TorA-expressing cells, there was a longer lag phase (∼10 h), but the resulting doubling time was again only slightly lower than the control (Figure 2B). These data show that TorA can functionally replace Hsp104 in protecting cells against heat stress.

Hsp104-mediated thermotolerance in yeast relies on ATP binding and hydrolysis, and the hydrolysis step can be inhibited *in vivo* by 3 mM GdnHCl [43,52]. As TorA also binds and hydrolyses ATP [3], we next determined whether a similar concentration of GdnHCl also inhibited the ability of TorA to functionally replace Hsp104 in the thermotolerance assay. The addition of 3 mM GdnHCl to the Hsp104-expressing cells when they were returned to 30°C cells post heat shock increased the lag phase and reduced the rate of growth (Figure 2A). The ability of TorA to rescue the growth of the *Δhsp104* cells following heat stress was also inhibited by 3 mM GdnHCl with the lag phase increased to 12 h and a reduced rate of growth (Figure 2B). A similar but less marked trend was also seen for the ΔN-TorA-expressing cells in the presence of 3 mM GdnHCl. These findings are consistent with low millimolar levels of GdnHCl-inhibiting TorA function *in vivo*. While it is conceivable that GdnHCl may have secondary effects on other ATPases required for protein refolding that are only evident in cells lacking Hsp104, we have previously shown that the loss of acquired thermotolerance in a *Δhsp104* is not further reduced by 3 mM GdnHCl [41].

Given that TorA was localised to the ER while ΔN-TorA was largely in the cytoplasm, the observation that both forms were able to complement the thermotolerance defect was surprising. However, in rat cells, TorA can translocate from the ER to the cytoplasm following an ER stress [8]. We therefore examined whether the 47°C heat stress applied here resulted in translocation of TorA from the yeast ER to the cytoplasm. Remarkably, TorA-GFP was found to completely re-localise to the cytoplasm after 2 h at 47°C, with the majority of the ΔN-TorA-GFP fusion also remaining in the cytoplasm (Figure 2C), although it appeared that both forms of TorA were excluded from the enlarged vacuoles in these heat-stressed cells.

We next examined the ability of TorA to protect Hsp104-deficient cells against a second form of environmental stress, high ethanol concentrations. The growth of *Δhsp104* cells is inhibited by 15% ethanol [31], and this behaviour was confirmed for the BY4741*Δhsp104* strain used here (Figure 2D). Expression of either Hsp104 or TorA in this *Δhsp104* mutant restored growth in 15% ethanol to a comparable degree (Figure 2D). This further demonstrated that TorA can functionally replace Hsp104 in protecting cells against a second, distinct environmental stress.

### TorA acts as an Hsp104-like chaperone *in vivo*

The ability of Hsp104 to protect cells against thermal and ethanol stress is directly related to its function as a molecular chaperone, breaking down protein aggregates arising as a consequence of stress-induced protein misfolding. Previous studies have shown that TorA, like Hsp104, can recognise and refold heat-denatured (i.e. misfolded) luciferase *in vitro*, in keeping with a molecular chaperone function [22]. To establish whether such chaperone activity could be detected *in vivo*, we exploited a *V. harveyi* luciferase-based assay used to demonstrate the chaperone function of Hsp104 from different yeast strains [46,47]. This *in vivo* protein refolding assay uses a heat-denatured *V. harveyi* luciferase fusion protein as a substrate. The basal luciferase activity is measured at 30°C representing the correctly folded luciferase prior to a short 46°C heat shock. The reactivation of the heat-denatured luciferase is then monitored over 4 h at 30°C in the presence of cycloheximide.

**Figure 3.**
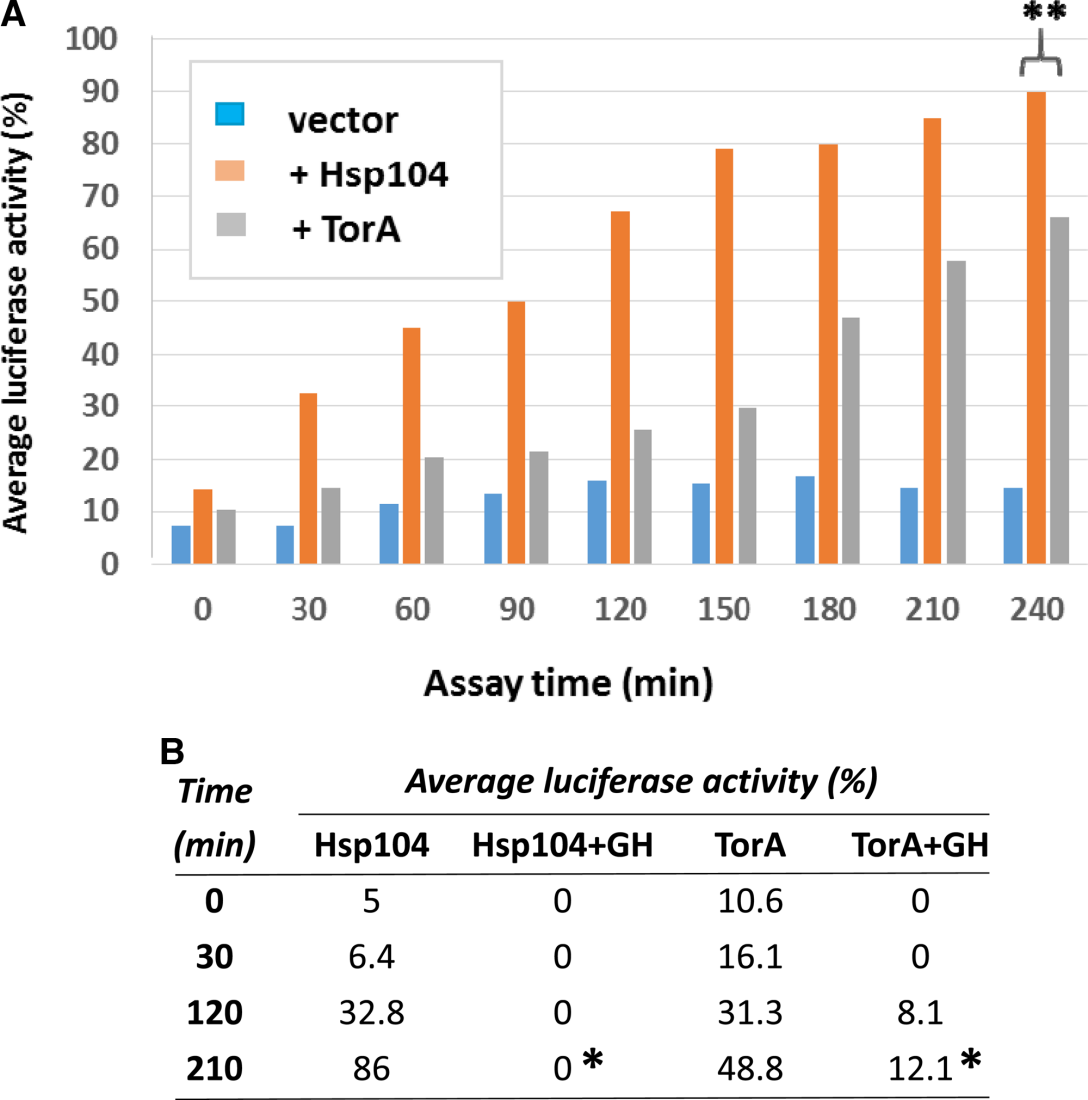
Human TorA can refold heat-denatured luciferase in a yeast strain lacking the molecular chaperone Hsp104. The Δ*hsp104* strain carrying the pGPDLuxAB(*HIS3*) plasmid was co-transformed with plasmids expressing either Hsp104 (+Hsp104) or TorA (+TorA) or the backbone plasmid pBEVY-U (vector). The reactivation of functional luciferase was assayed as described in Materials and Methods, and each value is the average of three technical replicates. (**A**) Reactivation for 4 h post transfer to 30°C. (**B**) The effect of the addition of 3 mM guanidine hydrochloride (+GH) on recovery of luciferase activity in the Δ*hsp104* strain expressing either Hsp104 or TorA. In all experiments, the data are based on the means of three independent experiments. * indicates that these levels of luciferase are significantly different (*P* ≤ 0.05) to the respective controls.

Δ*hsp104* strains expressing either TorA or ΔN-TorA were co-transformed with the plasmid pGDP-LuxAB expressing *V. harveyi* luciferase. In the Δ*hsp104* strain after the cells were returned to 30°C post heat shock, there was as expected minimal recovery of the luciferase activity, and even after 4 h, the levels only reached 15% of the pre-heat stress levels. In contrast, after 4 h, cells expressing Hsp104 showed almost 90% of the starting levels of functional luciferase (Figure 3A). Expression of TorA was also able to reactivate luciferase to significantly higher levels than observed in the control (*t* = 240 min; *P* ≤ 0.05), although there was an increased lag before maximal reactivation of luciferase was observed (Figure 3A). Such *in vivo* reactivation of a thermally denatured enzyme in the absence of Hsp104 is consistent with TorA having the ability to function as a molecular chaperone *in vivo*.

To establish whether or not the chaperone activity of TorA required ATP hydrolysis, the luciferase refolding experiment was repeated in the presence of 3 mM GdnHCl (Figure 3B). The ability of both Hsp104 and TorA to reactivate the luciferase in the presence of 3 mM GdnHCl was significantly reduced (*t* = 210 min; *P* ≤ 0.05) consistent with TorA requiring ATP hydrolysis to mediate refolding of the luciferase *in vivo*. These results further support the hypothesis that TorA has the properties of an ATP-dependent chaperone *in vivo*.

### Walker motif mutations in TorA affect on its chaperone activity *in vivo*

TorA has a single NBD with a non-canonical Walker A motif (GxxxxGKN) and a canonical Walker B motif (hhhhDE). The Walker motifs play an essential role in the function of other AAA+ ATPases including Hsp104 [29,53]. To further explore the role of TorA *in vivo*, we therefore generated several different mutations in these motifs and determined how these affected on the function and localisation of TorA expressed in yeast.

**Figure 4.**
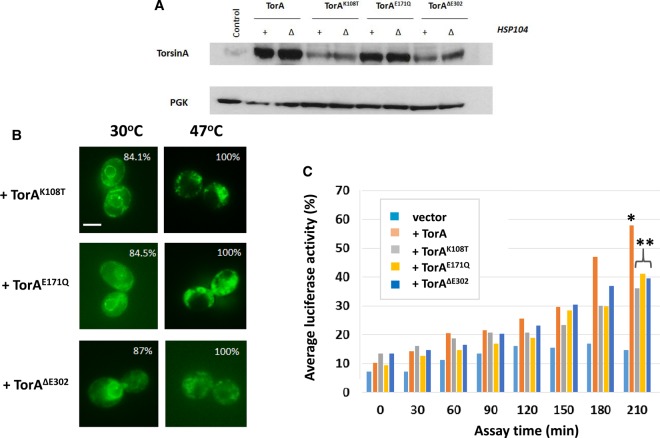
The effect of the K108T, E171Q and Δ302 mutations in TorA on expression, localisation and molecular chaperone activity *in vivo*. (**A**) Western blot analysis of the expression of the three TorA mutants K108T, E171Q and Δ302 in *HSP104*^+^ parent (+) and in the Δ*hsp104* strain (Δ). PGK is used as a loading control. (**B**) Localisation of the mutant TorA proteins fused to GFP in cells grown at 30°C or after exposure to 47°C for 2 h. The figures are the percentage of cells with the same phenotype as the cell shown in each respective panel. The magnification used was ×60 and the size bar is 2 μm. (**C**) The Δ*hsp104* strain carrying the pGPDLuxAB(*HIS3*) plasmid co-transformed with plasmids expressing either TorA (+TorA) or one of the three TorA mutants as indicated or the backbone plasmid pBEVY-U (vector). The data are based on the means three independent experiments. * indicates that these levels of luciferase are significantly different (*P* ≤ 0.05) to the vector-alone control. ** indicates that these levels of luciferase are significantly different (*P* ≤ 0.05) to the wild-type TorA control.

Single mutations were introduced into the Walker A motif (TorA^K108T^, unable to bind ATP) and B motif (TorA^E171Q^, can bind but not hydrolyse ATP) and expressed in yeast. Although the TorA^K108T^ mutant was expressed at a lower steady-state level than the wild-type TorA, this was not so for TorA^E171Q^ mutant (Figure 4A). Zacchi et al. [27] have previously reported that the TorA^K108T^, but not the TorA^E171Q^, mutation leads to an increased rate of degradation of TorA. Neither mutation had an effect on TorA localisation with both mutant proteins, when fused to GFP, being largely detected in the ER at 30°C (Figure 4B). Likewise, when subject to 47°C heat shock, the mutant proteins like the wild-type TorA protein re-localised to the cytoplasm (Figure 4B). When expressed in a *Δhsp104* mutant, both TorA mutants showed a significantly reduced ability to refold heat-denatured luciferase compared with the wild-type TorA control (*t* = 210 min; *P* ≤ 0.05), although the measurable refolding activity was not completely eliminated (Figure 4C). These data further support the hypothesis that TorA acts as an ATP-dependent chaperone *in vivo*.

Nagy et al. [54] reported that mutating the canonical Walker A motif into a non-canonical one reduced the activity of ClpB, the bacterial orthologue of Hsp104, both with respect to acquired thermotolerance and protein refolding. To explore whether restoring the canonical Walker A motif in TorA would positively affect on its chaperone activity *in vivo,* we introduced the necessary mutation (i.e. N109T; giving GxxxxGKT) and examined the activity of the TorA^N109T^ protein *in vivo*. The TorA^N109T^ mutant did not affect the ability of TorA to refold luciferase (Supplementary Figure S1), and this may reflect an underlying mechanistic difference between TorA and ClpB in their function as chaperones.

### Analysis of the *in vivo* function of the ΔE302 mutant of TorA

The role of the C-terminal domain of TorA has been studied primarily in relation to a single glutamic acid deletion (positions 302–303; designated TorA^ΔE302^) that is associated with the disease torsion dystonia [3,16]. Despite a plethora of *in vitro* and *in vivo* studies in *Drosophila*, mammalian cells and in yeast, it is still unclear whether or not this mutation causes loss of function of TorA [17,21,55] or mislocalisation [7,14,27,56–61]. Under normal growth conditions, i.e. 30°C, we found that TorA^ΔE302^, although expressed at a lower steady-state level than the wild-type TorA, was still targeted to the yeast ER and capable of translocating to the cytoplasm following a thermal stress (Figure 4B). However, expression of TorA^ΔE302^ in the *Δhsp104* mutant resulted in a significant reduction in the ability to refold heat-denatured luciferase compared with wild-type TorA (*t* = 210 min; *P* ≤ 0.05) and with a level seen with the two ATPase mutants of TorA analysed (Figure 4C). This finding suggests a loss of chaperone function of TorA^ΔE302^ in our yeast *in vivo* model.

### TorA can eliminate specific prion conformers from yeast cells

In the functional studies described above, we examined the role of TorA in dealing with protein aggregates generated by thermal stress *in vivo*. Such protein aggregates are generally amorphous in nature, and because Burdette et al. [22] had shown that that TorA may only deal with a particular subtype of protein aggregate, we also investigated whether TorA was able to process the highly ordered amyloid aggregates generated by the yeast protein Sup35 that give rise to the cytoplasmically transmitted [*PSI*^+^] prion.

There are several different conformational variants of the [*PSI*^+^] prion that affect to different extents on the non-sense suppression phenotype associated with the presence of the prion. ‘Weak’ [*PSI*^+^] variants have unstable phenotypes, low levels of non-sense suppression, and show mitotic instability and higher molecular mass, detergent-resistant Sup35 aggregates. In contrast, ‘strong’ [*PSI*^+^] variants contain predominantly aggregated Sup35 and are phenotypically characterised by high levels of non-sense suppression [62]. These phenotypic differences reflect an underlying difference in amyloid conformation [63].

**Figure 5.**
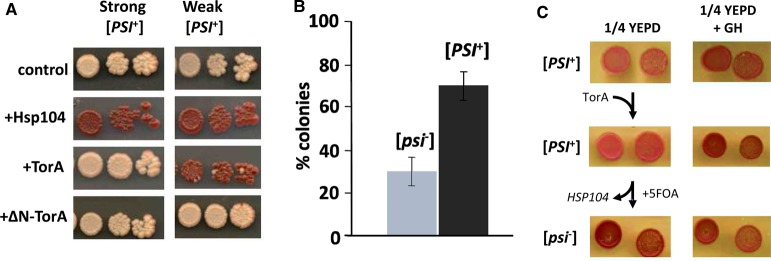
The impact of TorA expression on the maintenance of the [*PSI*^+^] prion. (**A**) Two different variants of the [*PSI*^+^] prion (‘strong’ and ‘weak’) in the *ade1-14* strain 74D-694 transformed with either pBEVY-U (control), pUKC2751 constitutively expressing Hsp104 (+Hsp104), pUKC2752 constitutively expressing TorA (+Tor) or pUKC2753, constitutively expressing ΔN-TorA (+ΔN-TorA). Three different cell dilutions are shown for each transformant, which are plated onto rich growth medium (1/4 YEPD). Red colonies are [*psi*^−^]. (**B**) 74D-694 carrying the weak [*PSI*^+^] was transformed with pUKC2752 (expressing TorA) and ∼400 independent transformants from each of two different transformation plates were transferred to 1/4 YEPD plates. After 3 days incubation, the number of red [*psi*^−^] colonies was counted and the total percentage was calculated for both plates. Each transfer by replica platting was performed twice for each plate and the standard deviation was calculated. (**C**) The [*PSI*^+^] strain YJW532 Δ*hsp104* carrying the plasmid pRS316*-URA3-HSP104* was transformed with plasmid pAG425GPD-ccdB-TorA-EGFP-*LEU2* (labelled TorA). Cells lacking pRS316*-URA3-HSP104* plasmid (labelled Hsp104) were then selected on 5-FOA. Two independent colonies at each stage were plated onto 1/4 YEPD to determine the [*PSI*^+^] phenotype and the presence of [*PSI*^+^] confirmed by growth on 1/4 YEPD + 3 mM GdnHCl.

Overexpression of Hsp104 eliminates the [*PSI*^+^] prion from cells [64], and so we investigated whether expression of TorA similarly affected the maintenance of the [*PSI*^+^] prion. As Hsp104 targets prion aggregates in the cytoplasm, we examined the localisation of TorA in both [*PSI*^+^] variants and found it to be predominantly localised to the cytoplasm in this strain (Supplementary Figure S2). This difference could reflect the fact that the 74D-694 strain is genetically distinct to the BY4741 strain used in our other studies. In a strong [*PSI*^+^] variant of the yeast strain 79D-694, no effect on the prion was observed, whereas in the weak [*PSI*^+^] variant examined, [*PSI*^+^] was eliminated by expression of TorA (Figure 5A). When the weak [*PSI*^+^] cells expressing TorA were plated onto YEPD, ∼30% of the transformed cells formed red [*psi*^−^] colonies (Figure 5B). Expression of truncated ΔN-TorA did not affect [*PSI*^+^] maintenance in either [*PSI*^+^] variant, suggesting that the ER targeting and glycosylation state are essential for [*PSI*^+^] elimination mediated by TorA. Similarly, expression of the ΔE302 mutant of TorA did not affect the maintenance of either [*PSI*^+^] variants consistent with it being a loss-of-function mutant (data not shown). Thus, TorA expressed in yeast eliminates the [*PSI*^+^] prion in a prion variant-specific manner, suggesting that TorA may only recognise particular amyloid conformations of Sup35.

The maintenance of the [*PSI*^+^] prion during cell division also depends on normal levels of Hsp104 [64], because the chaperone is required to fragment the Sup35 amyloid form to generate transmissible forms of Sup35, i.e. [*PSI*^+^] propagons. These are forms of the prion aggregate that can be passed on to daughter cells and hence propagate the prion state. We therefore determined whether TorA can functionally replace the prion maintenance function of Hsp104 *in vivo.* This was achieved using the strong [*PSI*^+^] strain YJW532, which carries a deletion of the *HSP104* gene, but also expresses wild-type Hsp104 from a centromeric plasmid with a *URA3* marker. A TorA-expressing plasmid pAG425GPD-ccdB-TorA-EGFP-*LEU2* was transformed into the YJW532 strain, and the resulting transformants were plated onto 5-FOA plates to select for Ura^−^ cells that had lost the Hsp104-expressing plasmid pRS316 [48]. Resulting Ura^−^ transformants were analysed for the [*PSI*^+^] phenotype of the YJW532 by plating onto 1/4 YEPD. None of the independent transformants examined showed a [*PSI*^+^] phenotype, demonstrating that TorA cannot maintain [*PSI*^+^] in the absence of Hsp104 (Figure 5C).

## Discussion

TorA is a member of the AAA^+^ ATPase superfamily of proteins, members of which share many structural properties, but which participate in a wide range of cellular functions [53]. Besides containing the motifs that define ATP binding and hydrolysis (i.e. Walker A and B and Sensor I and II motifs), TorA and other members of the family contain several other conserved domains. TorA is, however, considered an atypical member of the AAA+ ATPase family of proteins [6] for three reasons: (i) it has a non-canonical Walker A motif although as we show here (Supplementary Figure S1) this has no impact on its chaperone function *per se*; (ii) it lacks an arginine finger, the highly conserved catalytic arginine residue that is integral to the formation of the ATP-binding pocket [65] and (iii) it is the only AAA+ ATPase that resides in the ER. Yet, even though TorA was first described 20 years ago [3], there still remains considerable debate as to the normal cellular role(s) played by this glycoprotein in its various subcellular locations (see [6,66] for recent reviews).

Predominant among the AAA+ ATPase superfamily are molecular chaperone proteins that can remodel protein conformation or more complex protein structures. Molecular chaperones play an important role in maintaining protein homeostasis (for recent reviews, see [67]) by ensuring that misfolded proteins are removed before they can negatively affect on cellular functions and, consequently, cell viability. Failure of the molecular chaperone network and the resulting breakdown in protein homeostasis can have widespread ramifications including the emergence of neurological and neurodegenerative diseases [68,69]. The TorA-linked dystonia DYT1 is one such neurological disorder, yet how the functional impairment of TorA leads to the characteristic involuntary muscle movements and ultimately paralysis is poorly understood or indeed whether this is a result of a breakdown in protein homeostasis.

Given its sequence relationship to two members of the AAA+ ATPase family that have well-defined roles as molecular chaperones, i.e. Hsp104 and ClpB, it is plausible that a defect in TorA activity results in a breakdown of neuronal proteostasis. By exploring the chaperone-related activities of mammalian TorA in *S. cerevisiae*, we have tested this hypothesis. As demonstrated by two previous studies using yeast to explore different aspects of human TorA function [26,27], this highly tractable organism offers many advantages for both exploring TorA function, but also as a research platform for the wider study of protein homeostasis, chaperone function and the role of chaperones in certain proteinopathies [70,71].

Various lines of evidence have pointed to TorA acting as a chaperone that can participate in protein quality control specifically in relation to protein assembly in the ER [61,72]. These lines of evidence are either indirect or come from *in vitro* studies [e.g. 22]. Our study provides the first direct evidence that human TorA can function as an ATP-dependent molecular chaperone *in vivo.* We have done so by demonstrating that TorA can mediate the refolding of heat-denatured luciferase (Figure 3) and can rescue cells from stress-induced damage (Figure 2B,D). In both experiments, we used yeast cells lacking the ATP-driven disaggregase Hsp104 that is required for both activities in yeast, and thus, TorA appears to be able to functionally replace Hsp104 in this context.

That TorA can function in restoring protein function may seem surprising given that we have confirmed the findings of Zacchi et al. [27] that native TorA is localised to the yeast ER (Figure 1B) while Hsp104 is either in the cytoplasm or in the nucleus [73]. This conundrum is resolved by our finding that TorA re-localises to the cytoplasm following the heat stress applied during these assays, where it presumably is able to work on cytoplasmic, misfolded substrates. Such re-localisation has been reported for TorA in mammalian cells subject to ER stress [8], and a previous study in which TorA with its native signal sequence was expressed at high levels from a galactose-inducible promoter, TorA, was detected as large cytoplasmic aggregates [26]. Here, we expressed TorA from a lower efficiency constitutive *ADH1* promoter that may account for the discrepancy of our TorA localisation data and that of Zacchi et al. [27], with the findings reported by Valastyan and Lindquist [26].

Mutating either the Walker A or Walker B motifs in the single NBD of TorA significantly impaired the ability of TorA to reactivate heat-denatured luciferase, but did not completely ablate this activity (Figure 4C). Previous studies have shown that mutating the Walker A motif in NBD2 of Hsp104, while leading to a defect in ATP binding, nevertheless did not completely abolish the ability to reactivate heat-denatured GFP, while an equivalent mutation in NBD1 did ablate this activity [74]. This suggests a complex interplay between ATP binding, hydrolysis and protein remodelling by members of the AAA+ ATPase family and, as described above, TorA is considered to be an atypical member of this family and it is conceivable that it may act as a chaperone via a subtly different mechanism to that described for the archetypal members Hsp104 and ClpB [6].

That low millimolar concentrations of GdnHCl also impaired TorA function *in vivo* provides further evidence of the importance of TorA-mediated ATPase activity in reactivation of denatured luciferase (Figure 3B) and acquired thermotolerance (Figure 2B). GdnHCl directly inhibits the ATPase activity of Hsp104 without concomitant protein denaturation [43,52], but GdnHCl does not appear to act as a generic ATPase inhibitor. There have been reports that low millimolar levels of GdnHCl inhibit replication of both animal and plant positive-sense viruses [75] and, in poliovirus, this is a consequence of blocking ATP hydrolysis mediated by protein 2C, a nucleoside triphosphatase [76]. It remains to be established whether the inhibition of both TorA-mediated luciferase refolding and protection against thermal stress by GdnHCl is via the same mechanism as for Hsp104, but nevertheless having a chemical inhibitor of TorA function *in vivo* provides a powerful new approach to exploring TorA function in yeast and mammalian cells.

For Hsp104 and its bacterial orthologue, ClpB to function effectively as a disaggregase requires co-operation with the endogenous chaperone network. In particular, many studies have shown that Hsp70 (Ssa 1 in yeast) and Hsp40 (Sis1 in yeast) mediate substrate binding of Hsp104 and the subsequent activation of the chaperone function upon binding to protein aggregates [77–80]. For TorA to function as a disaggregase, one would therefore expect that it would need to functionally co-operate with the endogenous Sis1/Ssa1 chaperones, yet the interactions between Hsp70 and ClpB/Hsp104 are known to show a degree of species specificity [81,82]. This species specificity is mediated by the interaction of Hsp70 with the central coiled-coil-forming M region of ClpB/Hsp104 [83,84], a region absent from TorA. However, there have also been reports that Hsp104 can disaggregate proteins via an ATP-independent mechanism, for example, in its role in propagating the prion form of Sup35 in yeast [35]. It therefore remains to be seen whether the chaperone function of TorA we detect in yeast is Hsp70-dependent or -independent although TorA co-localises with Hsp70 to misfolded forms of α-synuclein in Lewy bodies [17]. It also remains to be established whether or not the chaperone activity we observe is similar to that reported for ClpB/Hsp104 [79]. What does emerge from our studies is that TorA can, in part, replace the function of Hsp104 in rescuing yeast cells from physical or chemical stress most likely as a consequence of its chaperone-associated properties.

One cellular role of Hsp104 that could not be replaced by TorA was to maintain the [*PSI*^+^] prion in dividing cells (Figure 5C), despite its cytoplasmic localisation (Supplementary Figure S2). Hsp104 is essential for the propagation of [*PSI*^+^] and all other yeast prions through its ability to fragment the amyloid polymers into smaller transmissible forms of the prion aggregates, i.e. transmissible propagons. Yet intriguingly, overexpression of Hsp104 also eliminates the [*PSI*^+^] prion — but no other prions — from growing yeast cells [64]. This effect was originally believed to be a consequence of the increased fragmentation activity of the chaperone, but our recent studies have suggested an alternative mechanism, namely that prion loss arises because of malpartition of the [*PSI*^+^] propagons between mother and daughter cells in cells with elevated levels of Hsp104 leading to increased retention of the propagons by the mother cell [85]. This could be related to a non-productive interaction between Hsp104 and the M region of Sup35 [77,86]. Whether TorA is able to directly interact with Sup35 in the cytoplasm (Supplementary Figure S2) via this site remains to be established although Frederick et al. [86] show that the dynamics of the interactions between chaperones and the Sup35 amyloid form very much depends on the structure of the amyloid core. It is therefore perhaps significant that we have found that TorA expression only affects weak [*PSI*^+^] variants, which have a structure that is more robust and resistant to fragmentation by Hsp104, although it remains to be established whether loss of the weak variant of [*PSI*^+^] from the TorA-expressing cells is due to increased retention of the Sup35 aggregates by the mother cells or via some other mechanism. Laudermilch and Schlieker [66] have recently suggested that TorA may act as a ‘holder chaperone’ and controls the tempo-spatial localisation of TorA and its associated factors.

Our data suggest that TorA has an ATP-driven function *in vivo* in yeast, yet there are several reports that TorA is inactive as an ATPase unless one of two cofactors are present. These cofactors are LAP1 and LULL1, with the latter being associated with the ER membrane [87–89]. Both LAP1 and LULL1 are potent activators of TorA-mediated ATP hydrolysis, yet *S. cerevisiae* has no orthologues of either cofactor. Nevertheless, that our data show that the chaperone properties of TorA require ATP hydrolysis suggests that one of many co-chaperones that activate the ATPase activity of other chaperones, e.g. Sti1 that activates the ATP hydrolysis activity of Hsp70 [90], may provide this function. There have been no reports, to date, of co-expression of either LULL1 or LAP1 with TorA in yeast although the expression of another TorA protein, called printor, had no measurable impact on TorA function in yeast [26]. Thus, the yeast TorA expression system we have established will allow us to further explore how the ATPase activity of TorA may be regulated and, by so doing, provide insights into a major human neurological disorder.

## Abbreviations

5-FOA: 5-fluoroorotic acid
AAA+: ATPase associated with diverse cellular activities
BiP: binding immunoglobin protein
ClpB: caseinolytic peptidase B
DYT1: dystonia type 1
ER: endoplasmic reticulum
ERAD: endoplasmic reticulum associated protein degradation
GdnHCl: guanidine hydrochloride
GFP: green fluorescent protein
Hsp104: heat shock protein 104
LAP1: lamina-associated polypeptide
LULL1: luminal domain-like LAP1
NBD: nucleotide-binding domain
PGK: phosphoglycerate kinase
TorA: TorsinA
YNB: yeast nitrogen base
ΔN-TorA: TorsinA lacking the N-terminal signal sequence
