# Supplementary material for: Human TorsinA can function in the yeast cytosol as a molecular chaperone
Source: Biochem J. 2017 Oct 5;474(20):3439–54. doi: 10.1042/BCJ20170395 (PMC5628414; doi:10.1042/BCJ20170395)
Supplement: Supplementary Table and Figures [file BCJ-474-3439-s1.pdf]

SUPPLEMENTARY INFORMATION

Table S1: Oligonucleotide primers used in this study

Primer Number	Primer Sequence (5' to 3')
hDYT1-Fwd	ACGTACGGATCCATGAAGCTGGGCCGGGCCGTG
hDYT1-Rev	ACGTACGTCGACTCAATCATCGTAGTAATAATC
Δ302TorA	TGTAAGCAGAGTGGCTGAGATGACATTTTTCCCC
Δ302TorAanti	GGGGAAAAATGTCATCTCAGCCACTCTGCTTACA
Tor1A_E171Q	CTGTGCGAGGTCCATCTTCATATTTGATCAGATGGATAAGATGCATG
Tor1A_E171Qanti	CATGCATCTTATCCATCTGATCAAATATGAAGATGGACCTCGCACAG
TorsinAK108T	GGTGGACAGGCACCGGCACGAATTTCGTCAGCAAGATC
TorsinAK108Tanti	GATCTTGCTGACGAAATTCGTGCCGGTGCCTGTCCACC
TorsinAN109T	GGACAGGCACCGGCAAAACTTTCGTCAGCAAGATC
TorsinAN109Tanti	GATCTTGCTGACGAAAGTTTTGCCGGTGCCTGTCC
attB1TorA-Fwd	GGGGACAAGTTTGTACAAAAAAGCAGGCTTCATGAAGCTGGGCCGGGCCGTGCT
attB2TorA-Rev	GGGGACCACTTTGTACAAGAAAGCTGGGTCATCATCGTAGTAATAATCTAACT
Hsp104attB1-Fwd	GGGGACAAGTTTGTACAAAAAAGCAGGCTTCATGAACGACCAAACGCAATTTACA
Hsp104attB2-Rev	GGGGACCACTTTGTACAAAGCTGGGTCATCTAGGTCATCATCAATTTC
ΔTAaatB1-Fwd	GGGGACAAGTTTGTACAAAAAAGCAGGCTTCATGGCTGGGCCTGCTGCTGCTGGCGCC

Supplementary Figure S1 Adam

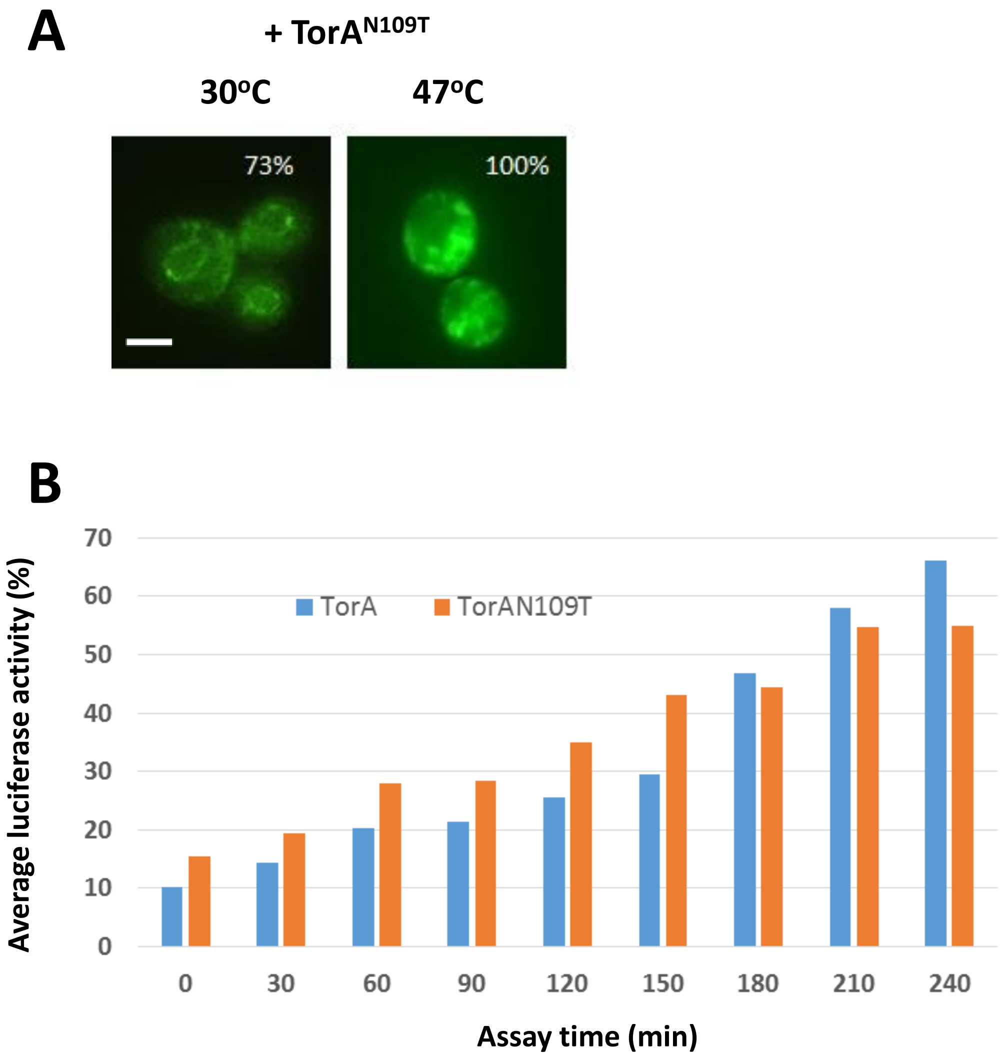

Figure S1: Analysis of the localisation and function of the N109T mutant of TorsinA. (A) Localisation of a TorA^{N109T}-GFP fusion in cells at 30ºC and after a 47ºC heat shock. The % cells with the phenotype shown in the panel are indicated. The magnification used was x60 and the size bar is 2µm.
(B) Reactivation of heat-denatured luciferase in a strain lacking Hsp104 comparing activity of the mutant TorA with wild type TorA.

Supplementary Figure S2
Adam

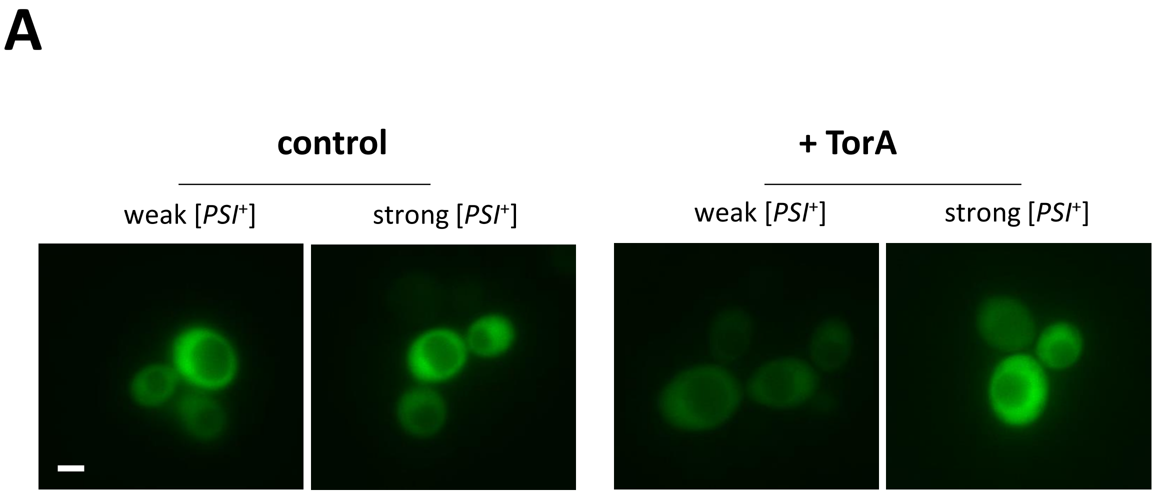

Figure S2: Localisation of human TorsinA expressed in the strain 74D-694 carrying either a weak or string variant of the [*PSI*$^+$] prion.
Localisation of a TorA-GFP fusion in weak and strong [*PSI*$^+$] cells at 30°C. The GFP vector was used as a control. The magnification used was x100 and the size bar is 2µm.
